# Comment on “Combating colorectal lung metastases via systemic therapy and thermal ablation: RAS mutation impact”

**DOI:** 10.1097/JS9.0000000000004741

**Published:** 2026-01-21

**Authors:** Xiaoxue Deng, Gantang Zhang, Limei Gao

**Affiliations:** a Gastrointestinal Surgery, Shenzhen Longhua District Central Hospital, Shenzhen, People’s Republic of China; b Nursing Department, Shenzhen Longhua District Central Hospital, Shenzhen, People’s Republic of China;

*To the Editor,*

Fan *et al*[1] report an impressive multicenter retrospective–prospective cohort of 387 patients with colorectal lung metastases (CRLM) treated with image-guided thermal ablation (IGTA) and systemic therapy, demonstrating that Kirsten rat sarcoma viral oncogene homolog (KRAS) and neuroblastoma RAS viral oncogene homolog (NRAS) mutations independently predict inferior local tumor control, progression-free survival, and overall survival. These real-world data support genotype-guided integration of local and systemic treatment in oligometastatic colorectal cancer.

The authors should be commended for assembling harmonized protocols across 12 interventional oncology centers and reporting clinically relevant endpoints, including local tumor progression-free survival and nomograms incorporating RAS status and tumor size[1]. Their observation that maximum lesion diameter ≥20 mm and higher lesion count are associated with worse outcomes, and that bevacizumab appears superior to cetuximab in KRAS-mutant disease, while the opposite holds in RAS wild-type patients, is highly relevant for multidisciplinary decision-making regarding ablation eligibility and peri-ablative systemic therapy sequencing. These results complement prior work showing that adequate ablation margins and careful patient selection can yield long-term local control in colorectal liver metastases^[2,3]^.

From a biological perspective, the finding that RAS-mutant tumors experience both higher local recurrence and poorer systemic control is consistent with experimental and clinical data linking activated RAS signaling to increased invasive potential, angiogenesis, and microscopic extension beyond the visible tumor margin[3]. These features provide a plausible explanation for the higher local tumor progression rate and worse survival in the RAS-mutant group.

We agree with the authors’ acknowledgment that, given the observational design and heterogeneous regimens, causal relationships between specific drugs and survival should be interpreted with caution[1]. However, we suggest further clarification on the following points to reduce residual confounding:

First, within each RAS stratum, patients receiving cetuximab, bevacizumab, or no biologic may have differed systematically in baseline risk (e.g. age, performance status, extra-pulmonary metastases, tumor burden, or line of therapy). Table 1 shows that RAS-mutant patients overall had slightly larger lung lesions and more frequent bevacizumab use, but targeted-agent subgroups are not described separately[1]. Clarifying whether bevacizumab-treated KRAS-mutant patients also had fewer or smaller lesions, less extra-pulmonary disease, or more intensive chemotherapy backbones than those given cetuximab would help readers judge to what extent confounding by indication might contribute to the striking difference in median overall survival (53.9 vs 18.0 months). An additional multivariable Cox model or propensity-weighted comparison directly contrasting bevacizumab – versus cetuximab-based regimens within the KRAS-mutant cohort could quantify whether this survival advantage persists after further balancing.

Second, systemic therapy and IGTA were integrated in various sequences, yet all time-to-event analyses were anchored at the date of ablation^[1,4]^. Classifying individuals according to the “ever used” targeted agent across the entire treatment course could introduce immortal-time and time-dependent confounding. A landmark analysis (e.g. at 3 or 6 months after IGTA) or sensitivity analysis treating targeted therapy as a time-varying covariate might reassure readers that the reported interaction between RAS status and biologic choice is not driven by differences in exposure timing.

Third, because local tumor progression was evaluated as a distinct endpoint, it would be informative to know whether the apparent benefit of bevacizumab in KRAS-mutant patients is primarily mediated through improved systemic disease control or whether any signal exists for enhanced local control around the ablation zone[1]. Reporting cumulative incidence curves for local tumor progression by targeted agent within the KRAS-mutant subgroup, or presenting an interaction term between RAS status, targeted therapy, and maximum tumor diameter in the local progression models, could help clarify whether intensified systemic therapy meaningfully compensates for the adverse ablation biology associated with RAS mutation.

These suggestions, if addressed using the existing dataset, could further strengthen the inference that RAS-stratified selection of cetuximab versus bevacizumab modifies outcomes in CRLM patients undergoing IGTA and help translate this work into genotype-guided peri-ablative treatment algorithms for future prospective evaluation.

## Data Availability

Not applicable.

## References

[1] Fan H Xu T Zeng Y. Combating colorectal lung metastases via systemic therapy and thermal ablation: RAS mutation impact. Int J Surg 2025. 10.1097/JS9.0000000000004089 41731873

[2] Hasegawa T Takaki H Kodama H. Three-year survival rate after radiofrequency ablation for surgically resectable colorectal lung metastases: a prospective multicenter study. Radiology 2020;294:686–95. 31934829 10.1148/radiol.2020191272

[3] Calandri M Yamashita S Gazzera C. Ablation of colorectal liver metastasis: interaction of ablation margins and RAS mutation profiling on local tumour progression-free survival. Eur Radiol 2018;28:2727–34. 29417253 10.1007/s00330-017-5273-2

[4] Rhaiem R Rached L Tashkandi A Bouché O Kianmanesh R. Implications of RAS mutations on oncological outcomes of surgical resection and thermal ablation techniques in the treatment of colorectal liver metastases. Cancers (Basel) 2022;14:816. 35159083 10.3390/cancers14030816 PMC8834154

